# Can the Epstein–Barr Virus Play a Role in the Development of Prostate Cancer?

**DOI:** 10.3390/cancers16020328

**Published:** 2024-01-11

**Authors:** Jacek Kiś, Magdalena Góralczyk, Dominika Sikora, Ewa Stępień, Bartłomiej Drop, Małgorzata Polz-Dacewicz

**Affiliations:** 1 1st Clinical Military Hospital with Outpatient Clinic in Lublin, 20-049 Lublin, Poland; jacekkis@gmail.com; 2 Department of Virology with Viral Diagnostics Laboratory, Medical University of Lublin, 20-093 Lublin, Poland; dominika.sikora@umlub.pl (D.S.); ewa.stepien@umlub.pl (E.S.); malgorzata.polz-dacewicz@umlub.pl (M.P.-D.); 3 Department of Medical Informatics and Statistics with e-Health Lab, Medical University of Lublin, 20-090 Lublin, Poland; bartlomiej.drop@umlub.pl

**Keywords:** prostate cancer, EBV, EBVCA, EBNA1

## Abstract

**Simple Summary:**

Prostate cancer (PCa) is a serious men’s health problem worldwide. Some authors report the occurrence of the Epstein–Barr virus (EBV), a well-known oncovirus, in PCa. Therefore, in this study, PCa tissues were screened for the presence of EBV. Then, the frequency and titer of antibodies against EBV in the serum of EBV-positive and EBV-negative patients were compared. The results obtained showed a higher frequency and level of Epstein–Barr virus capsid antigen (EBVCA) and Epstein–Barr nuclear antigen 1 (EBNA1) antibodies in patients with EBV-positive PCa. Higher levels of tested antibodies were observed in more advanced stages of the disease (high-risk group, high Gleason score (GS) and stage T2). These observations may suggest a role for EBV in the development and/or progression of PCa.

**Abstract:**

Prostate cancer (PCa) is the fourth most frequently diagnosed cancer worldwide, accounting for 7.3% of all cancers. PCa mortality is the fifth most common cause of cancer death. Despite well-known factors influencing the development of PCa, such as age, race/ethnicity and family history, many researchers have raised the possibility of persistent infections with oncogenic viruses. Therefore, we aimed to assess the frequency of Epstein–Barr virus (EBV) DNA in tissue collected from PCa patients. Next, the frequency and the level of Epstein–Barr virus capsid antigen (EBVCA) and Epstein–Barr nuclear antigen 1 (EBNA1) antibodies in both IgA and IgG classes were measured. The antibody titer was also analyzed depending on the risk group, Gleason score (GS) and tumor, node, metastasis (TNM) classification. Serum samples were analyzed using the Microblot-Array EBV IgM, IgA and IgG test kits. The study group consisted of 115 patients diagnosed and histopathologically confirmed with PCa. In 49% of patients included in the study, EBV DNA was detected in the tumor tissue. The studies showed both higher seroprevalence and higher antibody titers in patients with EBV-positive PCa compared to patients with EBV-negative PCa. We also observed a dependence of antibody titer on pathological features, such as GS, risk group and T stage.

## 1. Introduction

Cancer is a serious public health problem on a global scale. According to the World Health Organization estimates, the number of newly detected cancer cases shows an increasing tendency and is expected to reach 30.2 million cases in 2040 [1].

The incidence and mortality trends of malignant neoplasms in Poland have been observed for many years. These trends are determined by both the age structure of the population and alterations in the population’s exposure to carcinogens. In 2020, the National Cancer Registry (NCR) reported more than 146,000 new cancer cases and nearly 100,000 cancer deaths. Malignant neoplasms are the second leading cause of mortality in Poland, accounting for 21.8% of deaths among males in the same year.

Prostate cancer (PCa) poses a significant worldwide health issue for men, being the fourth most frequently diagnosed cancer globally [2]. According to GLOBOCAN 2020, 1,414,259 new cases of PCa were reported in 2020, which corresponded to 7.3% of all cancer cases. Excluding non-melanoma skin cancer, PCa mortality is the fifth leading cause of cancer deaths in the world [3]. It is caused by an indolent course of disease and symptomless early stage of cancer [4]. PCa is estimated to account for 3.8% (375,304 men) of all cancer deaths in the male population [3].

Analyzing the data for Poland, a systematic increase in the incidence of PCa has been observed and further growth can be expected. The number of cases is supposed to reach 20,000 per year [5]. The Polish NCR reported that, in 2020, the incidence of PCa was 19% (14,404 men) with deaths caused by the disease at 11% (5748 men) [6]. This type of cancer has displayed the highest incidence rate with a mortality plateau in the first decade of the 21st century. However, since 2004, there have been indications of its increased prevalence. In 2020, there were nearly 4000 fewer new cases than in 2019, but fatalities from cancer increased by 130 [6]. The reduction in the incidence of malignant tumors observed in 2020 is most likely related to the COVID-19 pandemic, resulting from restrictions imposed in connection with the pandemic, such as the transformation of hospitals into infectious disease units and the limited availability of specialists.

Many factors are undoubtedly involved in the development of PCa: age, race/ethnicity and family history [7]. Moreover, smoking, alcohol abuse and dietary factors are also associated with the development and progression of PCa [8]. On the other hand, many authors draw attention to the role of persistent viral infection in the development of PCa [9,10,11].

Almost 20% of cancers can be attributed to viral infections, and 12% of these cases are caused by oncogenic viruses [12]. Oncoviruses can act as direct carcinogens, where viral oncogenes directly contribute to the transformation of cancer cells, or as indirect carcinogens, where virus-induced infection leads to carcinogenic mutations [13]. According to the International Agency for Research on Cancer (IARC), there are seven viruses with oncogenic potential. This group includes DNA viruses: Epstein–Barr virus (EBV), Kaposi’s sarcoma-associated herpesvirus (KSHV), hepatitis B virus (HBV), human papillomavirus (HPV) and Markel cell polyomavirus (MCPyV) and RNA viruses: human T-lymphotropic virus 1 (HTLV-1) and hepatitis C virus (HCV) [12,14]. Studying other viruses may provide new information that will impact cancer prevention and treatment.

EBV, also known as human herpesvirus 4 (HHV4), was discovered by Michael Epstein and Yvonne Barr in 1964 while working with lymphoblast cultures from patients with Burkitt lymphoma (BL) [15]. EBV, a member of the Herpesviridae family, an enveloped virus with double-stranded DNA genome, is widespread in the human population where over 90% have antibodies against this virus. It has been classified as a Group 1 carcinogen by the International Agency for Research on Cancer (IARC), the cancer research agency of the World Health Organization (WHO) [16].

Saliva is the primary mode of transmission for EBV [17]. In order to establish enduring persistence in memory B-lymphocytes, EBV has devised efficacious methods of multiplying within oropharyngeal epithelial cells and mucosal B-lymphocytes. While the main targets of EBV remain epithelial cells and B-lymphocytes, the virus can also infect natural killer cells, follicular dendritic cells and T-lymphocytes [15]. The virus can also be transmitted through organ transplantation and blood transfusion. Immunocompromised elderly individuals are particularly vulnerable to the harmful effects of EBV.

EBV infection most often occurs in early childhood and is usually mild and self-limiting. It is the main etiological agent of infectious mononucleosis, which manifests as fever, enlarged and sore lymph nodes and pharyngitis [18]. In addition, some case reports as well as epidemiological studies indicate an association of EBV infection with diseases such as lymphoproliferative disorders, systemic lupus erythematosus, vitamin D deficiency, chronic fatigue syndrome, thyroid disorders, rheumatoid arthritis (RA), multiple sclerosis (MS) as well as other autoimmune disorders [19].

Like other viruses belonging to Herpesviridae family, EBV has the ability to establish latency in the cells of the infected organism, which may periodically reactivate to the lytic phase [20].

The association of EBV with the development and progression of various B-cell cancers, e.g., BL, Hodgkin’s lymphoma (HL), but also epithelial cancers such as gastric cancer (GC) and nasopharyngeal cancer (NPC) has already been established [21,22,23,24]. Moreover, EBV has also been detected in oral cancer, breast cancer and cervical cancer (CC), although its role in the development of these cancers is controversial [25].

In the scientific literature, some researchers raise the issue of the possible role of EBV in the development of PCa [26].

Therefore, we aimed to assess the frequency of EBV DNA in tissue collected from PCa patients. Next, the frequency and the level of Epstein–Barr virus capsid antigen (EBVCA) and Epstein–Barr nuclear antigen 1 (EBNA1) both in IgA and IgG classes were measured. The antibody titer was also analyzed depending on the risk group, Gleason score (GS) and tumor, node, metastasis (TNM) classification.

## 2. Materials and Methods

### 2.1. Characteristics of Patients

The study enrolled 115 male patients diagnosed and histopathologically confirmed with PCa. Patients were hospitalized at the Urology Department of the 1st Military Clinical Hospital with Outpatient Clinic in Lublin from January 2023 to November 2023. Exclusion criteria for the study were patients who received chemotherapy or radiotherapy treatments. All patients underwent radical prostatectomy. The mean age of the patients was 68.9 ± 7.4.

Table 1 presents the risk classification according to the European University Association (EUA), which divides patients with PCa into three groups: the low risk, the intermediate risk and the high risk based on three contributing factors: prostate-specific antigen (PSA) level, GS and TNM staging system [27,28,29,30]. Patients were assigned to their respective groups based on this classification.

The characteristics of the subjects are presented in Table 2. Detailed patient characteristics also included EBV test results. Patients were categorized into two groups: EBV-positive (49.57%) and EBV-negative (50.43%). Both groups did not differ significantly in terms of demographic and social characteristics. However, they differed significantly in terms of pathomorphological features, i.e., risk group (according to the EAU classification: low, intermediate and high), GS and TNM classification.

There were no cases of T3 and T4 in the study group. No regional lymph node metastasis (N0) as well as no distant metastasis (M0) were diagnosed.

### 2.2. Sample Collection

The clinical material for research consisted of serum and fresh-frozen tumor tissues (20 mg) collected from PCa patients. Samples were coded based on a sample identification system that ensured patient anonymity.

Venous blood samples (3–5 mL) were collected according to standard hospital procedure (blood was collected via venipuncture in tubes without anticoagulant). Blood was collected for routine testing, and the remaining blood samples were transferred from the hospital laboratory for our studies. Then, blood samples were centrifuged (1500× *g*/15 min at room temperature), and the serum was separated. Tumor tissue and sera were stored at −80 °C until analysis.

### 2.3. Isolation and Detection of EBV DNA

The fresh-frozen tumor tissues were cut and homogenized in a manual homogenizer Omni TH/Omni International/Kennesewa, GA, USA. DNA was extracted using QIAampDNA Mini Kit (Qiagen, Hilden, Germany) as described in manufacturer’s protocol. To verify the quality of the obtained DNA (presence of inhibitors of Polymerase Chain Reaction (PCR)), a β-globin assay was performed. The isolated material was subsequently amplified using commercially available GeneProof Epstein–Barr virus (EBV) PCR Kit (Brno, Czech Republic). All samples and also a negative control were analyzed in duplicate. A specific conservative DNA sequence for the EBNA1 was amplified during the PCR process according to manufacturer’s protocol. The PCR was performed using LightCycler 2.0 Software Version 4.1. (Roche Applied Science System, Penzberg, Germany).

### 2.4. Detection of HPV

To detect and determine the HPV genotype, the commercially available INNO-LiPA HPV Genotyping Extra II assay/Fujirebio/Ghent/Belgium diagnostic kit was used. This kit is based on the amplification of a 65 bp fragment from the L1 region of the HPV genome using the SPF10 primer set. The PCR products were then typed using a reverse hybridization method.

### 2.5. Antibodies Detection—Serological Methods

Anti-EBV antibodies in the IgA, IgM and IgG classes were detected using the Microblot-Array EBV IgM, IgA and IgG test kit (TestLine Clinical Diagnostics s.r.o., Brno, Czech Republic). This test is dedicated for detection of IgA, IgG and IgM antibodies in human serum, plasma or cerebrospinal fluid and contains a selected combination of specific parts of EBV antigens, i.e., EBNA1, EBNA2, VCA p18, VCA p23, p54 Early Antigen D (EA-D p54), EA-D p138, EA-R, Rta, ZEBRA, gp85, gp350 and latent membrane proteins 1 (LMP1). The interpretation considers whether there is a reaction against at least one antigen, either EBNA1 or VCA p18. The results were provided in U/mL. Negative results were below 185 U/mL, borderline results were between 185 and 210 U/mL and positive results were above 210 U/mL. The test results were read and interpreted using Microblot-Array reader and software version 2.0.4. 

### 2.6. Statistical Analysis

Results were analyzed using GraphPad Prism 10 software version 10.1.0 (San Diego, CA, USA). Categorical variables were expressed as numbers and percentages. The evaluation of the distribution of continuous variables was assessed using the Shapiro–Wilk test. The baseline characteristics of patients was given as a percentage. Pearson’s chi-squared test and Fisher’s extract test were used to compare the frequency of antibodies in both groups. The Mann–Whitney test or Kruskal–Wallis test was used to compare antibody levels between two groups. The results were considered significant at *p* ≤ 0.05.

### 2.7. Ethics

The research was approved by the Medical University of Lublin Ethics Committee and was in accordance with the GCP regulations (no. KE-0254/194/10/2022, 6 October 2022). Written informed consent was obtained from each participant.

## 3. Results

### 3.1. Prevalence of EBVCA and EBNA1 Antibodies in IgA and IgG Classes (U/mL) among Patients with PCa

As a first stage, fragments of PCa tissue were tested for the presence of EBV and additionally for HPV. The results obtained showed the presence of EBV DNA in 49.57% of PCa patients. HPV was not detected.

The presence of IgM anti-EBV antibodies was not detected in any of the examined patients. Of the many antigens included in the diagnostic kit used, only antibodies against the two main EBV antigens were detected in the serum of the examined patients, i.e., anti-EBVCA and anti-EBNA, both in the IgG and IgA classes. Due to this fact, the frequency and titer of these antibodies in the serum of patients with EBV-positive and EBV-negative PCa were analyzed.

**Figure 1:**
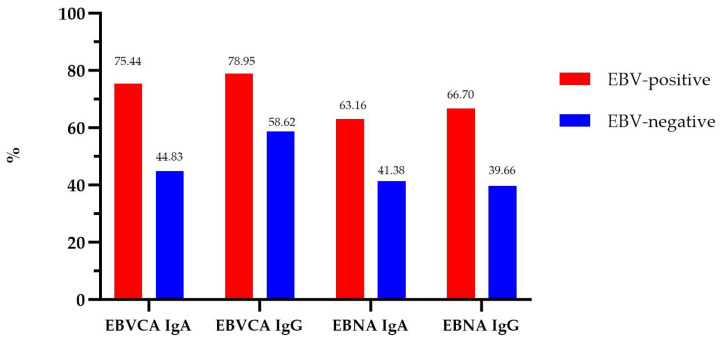
Prevalence of EBVCA and EBNA1 antibodies in PCa patients.

The results of the seroprevalence are presented in Figure 1. All antibodies were the most frequent in EBV-positive PCa patients. In EBV-positive patients, EBVCA antibodies of the IgA class were found in 75.44% and of the IgG class, they were found in 78.95%. Whereas, in the EBV-positive group, anti-EBNA1 antibodies of the IgA class were detected in 63.16% and of the IgG class, they were found in 66.70%.

In the next step, we analyzed the prevalence of studied antibodies according to the risk groups, GSs and T stages.

Table 3 presents an analysis of the frequency of antibodies in different risk groups. EBVCA IgA, EBNA1 IgA and IgG antibodies were found to be detected more frequently in the high-risk EBV-positive PCa patients. This difference was statistically significant. However, no such difference was observed in the EBV-negative group.

Table 4 presents the frequency of tested antibodies in relation to total GS. A higher percentage of patients with antibodies was observed in higher GSs, i.e., eight and nine in the EBV-positive group. A statistically significant difference in the frequency of antibodies depending on the GS was observed with EBNA1 IgA antibodies in EBV-positive patients.

Seroprevalence in relation to T stage was compared (Table 5). In the group of EBV-positive patients, higher seroprevalence was observed in patients with the T2 stage. EBVCA in the IgA class and EBNA in both the IgA and IgG classes were detected significantly more often.

### 3.2. Antibody Levels for EBVCA IgA and IgG and EBNA1 IgA and IgG in PCa Patients in Relation to Risk Group, GS and T Stage in EBV-Positive PCa Patients

In further analysis, we included only EBV-positive PCa patients. We evaluated the level of anti-EBVCA and anti-EBNA1 antibodies both in IgA and IgG classes in relation to the risk group, GS and T stage.

**Figure 2:**
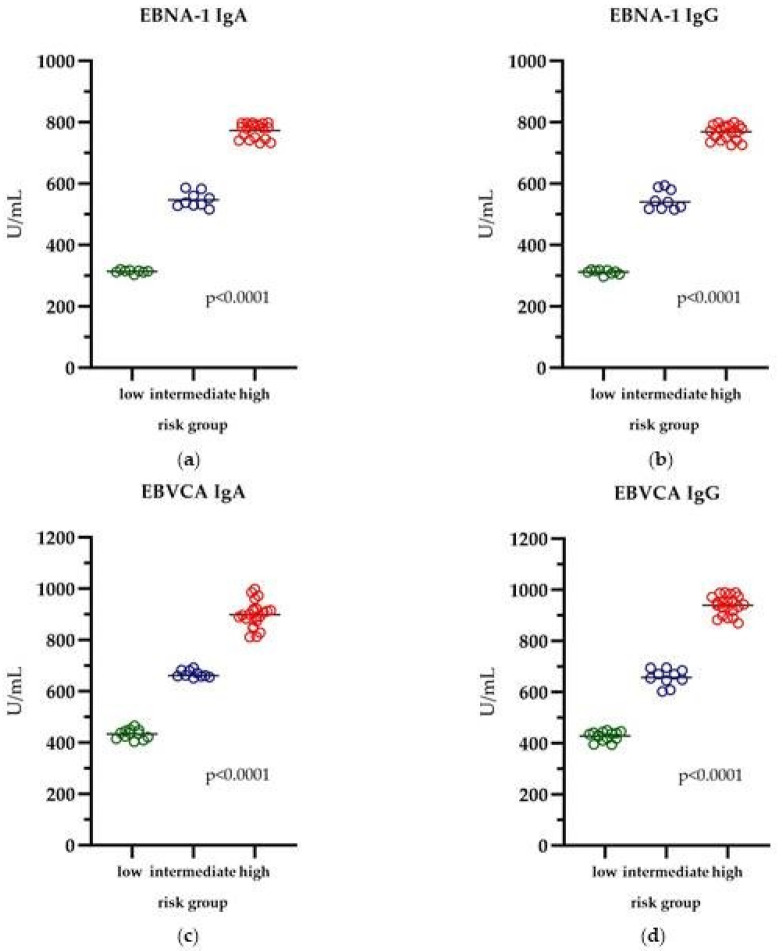
The level of antibodies in relation to the risk group: (**a**) EBNA1 IgA, (**b**) EBNA1 IgG, (**c**) EBVCA IgA and (**d**) EBVCA IgG; Kruskal–Wallis test. Green color—low risk group; Blue color—intermediate risk group; Red color—high risk group.

The level of EBVCA and EBNA1 antibodies in the IgA and IgG classes in PCa patients divided into the risk groups is shown in Figure 2. The highest level of all tested antibodies was observed in the high-risk group and was, respectively, EBNA1 IgA—783.1 U/mL (*p* = 0.0001) (Figure 2a), EBNA1 IgG—769.0 U/mL (*p* = 0.0001) (Figure 2b), EBVCA IgA—898.3 U/mL (*p* = 0.0001) (Figure 2c) and EBVCA IgG—942.7 U/mL (*p* = 0.0001) (Figure 2d). The differences between the levels of all tested antibodies depending on the risk group were statistically significant.

**Figure 3:**
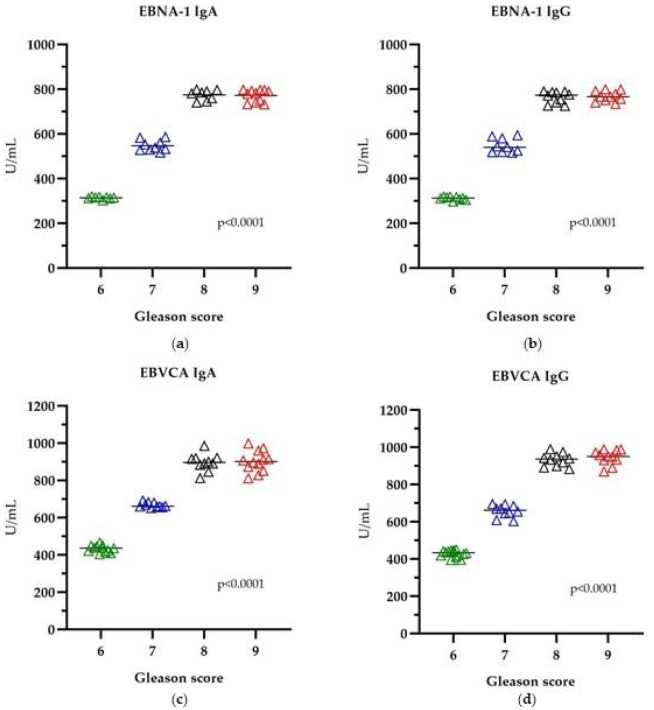
The level of anti-EBV antibodies in relation to GS: (**a**) EBNA1 IgA, (**b**) EBNA1 IgG, (**c**) EBVCA IgA and (**d**) EBVCA IgG; Kruskal–Wallis test. Green color—GS 6; Blue color—GS 7; Black color—GS 8; Red color—GS 9.

Figure 3 shows the level of analyzed antibodies in the group of EBV-positive patients with PCa. The highest antibody titer was found in patients with GSs of eight and nine (*p* < 0.0001).

**Figure 4:**
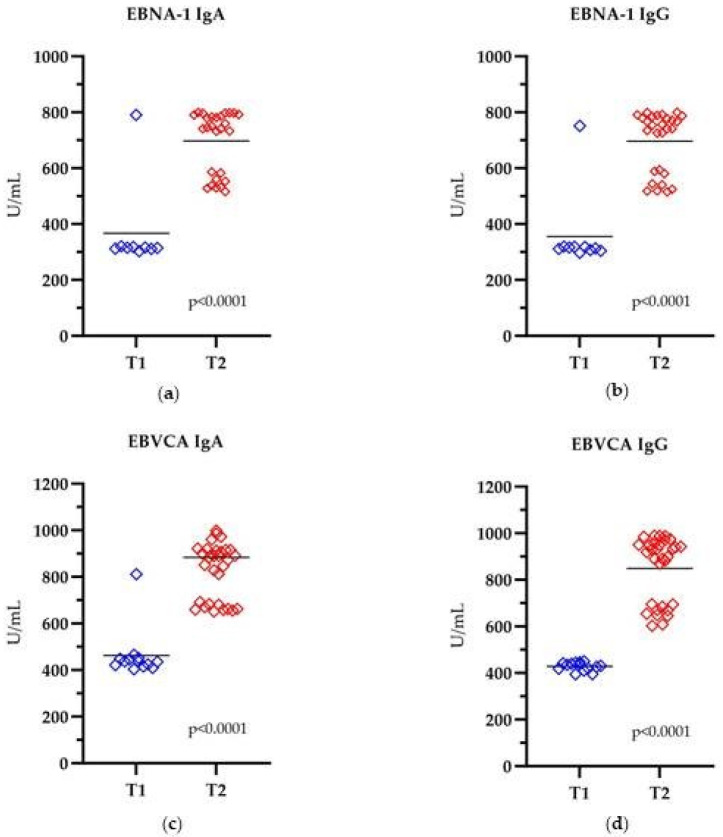
The level of anti-EBV antibodies in the relation to the T stage: (**a**) EBNA1 IgA, (**b**) EBNA1 IgG, (**c**) EBVCA IgA, (**d**) EBVCA IgG; Kruskal–Wallis Test. Blue color—T1; Red color—T2.

Analysis of the level of all antibodies in the EBV-positive group, categorized according to the T stage, shows that in the T2 stage, the antibody level is significantly higher compared to the antibody level in the T1 stage (Figure 4). Differences in the concentrations of all types of antibodies depending on the T stage are statistically significant (*p* < 0.0001).

### 3.3. EBV Viral Load in Relation to GS

Tissue from PCa patients was determined to have EBV viral load and, therefore, was classified as low or high based on the cycle threshold (Ct) value of the viral gene. The result was considered high when the Ct value of the viral gene was < 38 and low when the Ct value was ≥38. A high level of viral load was detected in 66.7% of patients with 8–9 GSs, while in patients with 6–7 GSs, a high level was detected in only 33.3% of the subjects. This difference was statistically significant (*p* = 0.0083) (Table 6).

## 4. Discussion

Many pathogens often occur in prostate tissues, which can cause chronic inflammation and, consequently, the development of PCa [31,32]. Chronic inflammation can be caused by various pathogens such as human papillomavirus, herpes simplex, Epstein–Barr virus, Cutibacterium acnes, Neisseria gonorrhea and Mycoplasmas [33,34].

Although studies have shown the detection of EBV in prostate tissue, the role of EBV in the development of PCa is still unclear. Several research groups have reported high rates of EBV detection in PCa tissue. Nahand et al. [35] identified EBV in 49.3% of PCa specimens from Iranian residents. Likewise, a study of Australian men using in situ PCRs reported EBV detection in 40% of PCa tissue [36]. Ahmed et al. [26], examining prostate tumor tissue samples from patients in Pakistan, found a high percentage of EBV-positive samples—39.39%. Additionally, in the US, another study using the immunochemistry method detected EBV infection in almost 37% of patients with PCa [37]. Our results are similar to those mentioned above. On the other hand, in the Swedish population, no presence of EBV was detected in PCa tissue [38]. Some authors assume the possibility of EBV infection through sexual contact, which may consequently lead to the development of PCa [39,40].

Other researchers have observed co-infections in PCa patients involving two viruses, and there is an increasing amount of research that is investigating the association with co-infection oncogenic viruses. The most prevalent co-infection in PCa is EBV/HPV co-infection. An examination of the findings by Whitaker et al. [36] established that the existence of co-infection could potentially impact cancer advancement. It was observed that EBV/HPV co-infection was notably prevalent in patients with PCa (55%) compared to individuals with benign PCa (15%) and those with a normal prostate (30%). Additionally, a separate study involving 67 patients with PCa discovered that EBV/HPV co-infection was present in 14.9% of participants [35]. However, we did not detect any cases of EBV/HPV co-infection in our study group.

It is known that the mere presence of EBV DNA cannot be the only evidence of its role in the development and progression of this cancer. Therefore, we assessed the prevalence and level of antibodies against the main antigens of the EBV, i.e., EBVCA and EBNA1, both in IgA and IgG classes, depending on the clinicopathological parameters such as GS, risk group and tumor size (T). We observed higher GSs in EBV-positive PCa patients compared to EBV-negative individuals. Similar results were obtained by other authors comparing histopathological parameters expressed by the GS [26].

EBV has a number of defense mechanisms that allow it to evade the host’s immune system. Therefore, neither the humoral nor cellular response eliminates the virus from infected cells, which causes it to remain in infected humans for life. After entering the host cell, the nucleocapsid is released into the cytoplasm and the genetic material is transported to the cell nucleus. The EBV life cycle consists of a lytic (productive) and a latent phase periodically reactivated into the lytic cycle, which plays an important role in the development of EBV-related cancers [41,42].

In the latent phase, EBV DNA occurs in the form of episomes in the cell nucleus [43,44]. More than 80 antigens are expressed in the EBV lytic cycle. However, in the latent phase, they are synthesized into a small number of latent viral genes, i.e., EBNAs, LMPs and noncoding RNAs: EBV-encoded small RNAs (EBERs) and microRNAs (miRNAs) in the BamHI-A rightward transcripts (BARTs) or the BHRF1 region.

Latency phase, depending on the gene expression profile, is divided into four types [45]. Different types of latency may occur in different cancers, examples of which are given in parentheses. Thus, in latency type 0, only EBERs are synthesized; in type I, only EBERs and EBNA1 (Burkitt lymphoma and gastric carcinoma) are synthesized; in type II, EBERs, EBNA1, LMP1 and LMP2 genes (some types of Hodgkin’s lymphoma, NPC, CAEBV and T/NK lymphomas) are expressed; and in type III, all genes of type II and additionally EBNA2, EBNA3 and EBNA-LP (most cases of PTLD or lymphoblastoid cell lines (LCLs)) are expressed. Moreover, BART miRNA expression is higher in latency types I and II, while higher BHRF1 miRNA expression levels are observed in latency type III [45].

Interesting results were presented by Ahmed et al. [26], analyzing the expression of EBV latency genes. Namely, they showed an atypical EBV latency profile (II/III) in PCa. On this basis, these authors suggest a relationship between EBV infection and the development of PCa [26].

Serological tests are very useful for detecting anti-EBV antibodies. Different stages of EBV infection (acute, reactivation and past) are characterized by the presence of different antibody profiles (IgA, IgG and IgM), which has been used in the diagnosis of many diseases associated with EBV infection. ELISA tests are commonly used in clinical practice to detect antibodies against various viral antigens, i.e., EBVCA, EA and EBNA. The detection of VCA IgM and VCA IgG in the absence of EBNA1 IgG indicates an acute infection, while the presence of VCA IgG and EBNA1 IgG and the absence of VCA IgM indicates a past infection [46].

Due to the lack of an effective vaccine to prevent persistent EBV infection, research is being carried out on various viral biomarkers that may have diagnostic and prognostic applications in diseases associated with EBV. One of the well-documented and widely studied cancers is nasopharyngeal cancer, which has a good prognosis if diagnosed early. An increased immune response to EBV indicates poor control of the virus, which promotes the development of various diseases, including cancer [47]. This applies especially to antibodies of the class IgA. Studies have shown that men with VCA IgA antibodies were approximately 22 times more likely to develop NPC [48]. The study conducted by Liu et al. [49] has shown that evaluation of EA-IgA, VCA-IgA and EBNA1-IgA antibodies is an effective method in NPC diagnosis. In turn, among patients with gastric cancer, antibodies against EBNA are detected in 99% of cases, and antibodies against capsid antigen are detected in 98% of cases regardless of tumor EBV status [50].

Our study showed a higher incidence of antibodies in EBV-positive PCa patients than in the EBV-negative group. In addition, antibody levels were also significantly higher. We also observed a dependence of antibody titer on pathological features, such as GS, risk group and T stage. It seems interesting that we did not detect antibodies indicating reactivation of the EBV infection, i.e., EA, Zta and Rta. This could indicate that only latent infection plays a role in PCa [51,52].

EBNA1 expression occurs in both latent and lytic phases of EBV infection. It is worth emphasizing that EBNA1 plays a dual role. On the one hand, it maintains latency, and on the other hand, it plays a role in virus reactivation and lytic infection [51,52].

We tried to assess the viral load and compare it with the Gleason score. Preliminary analysis showed that high levels of viral load were detected in 66.7% of patients with 8–9 GSs, while in patients with 6–7 GSs, they were detected only in 33.3% of those tested (*p* = 0.0083).

The limitation of our research is the group of patients is too small, which might lead to risk of some biased interpretations regarding the reactivation of the latent phase. The group of patients with detected EBV DNA consisted of only 57 individuals. This could be the reason why anti-EA and anti-Zta antibodies, indicating reactivation of the infection, were not detected in this group of patients. Therefore, these studies should be treated as preliminary. However, due to the fact that a significantly higher titer of antibodies, especially EBNA1, was observed in EBV-positive patients and their level correlated with more advanced clinical stages (it was significantly higher in Gleason scores of eight and nine), and a higher viral load in patients with a high Gleason score was also observed, these results encourage further in-depth research. However, we are still collecting clinical material to check the repeatability of the results in a larger group of PCa patients.

We realize that serological tests are not sufficient. We treat them as preliminary research. As mentioned above, in the latent phase, they are synthesized as a small number of latent viral genes, i.e., EBNAs, LMPs and noncoding RNAs: EBV-encoded small RNAs (EBERs) and microRNAs (miRNAs) in the BamHI-A rightward transcripts (BARTs) or the BHRF1 region [44]. Therefore, subsequent studies should focus on the assessment of other proteins. One viral oncoprotein of interest is BARF1, expressed in EBV-associated cancers encoded by the BamHI-A rightward frame 1 [53]. The secreted BARF1 (sBARF1) protein can promote cell growth, immortalization and tumor transformation through the NF-κB pathway. BARF1 can be detect during the lytic cycle in tumor cells. EBV-associated tumors show various expression patterns of latent viral genes. BamHI-A right frame 1 (BARF1) is expressed selectively, which can play a role in immune response [54]. As research shows, in EBV-positive GC, BARF1 is expressed in the absence of LMP1 [55], while in NPC, BARF1 may function as an oncogene, in parallel with LMP1 [56]. This may be an interesting subject for further research.

Moreover, an interesting issue would certainly be to assess whether the presence of the EBV in prostate tissues affects the survival of patients. However, it requires specially planned long-term research. It would be worth considering this study in the future.

EBV is a common virus associated with a significant number of cancers worldwide Due to the limited number of studies on the role of EBV in PCa, we hope that the presented results will shed new insights into the role of EBV in promoting carcinogenesis in PCa. However, further research is necessary, on the one hand, to thoroughly understand the impact of the EBV on the development and progression of PCa and, on the other hand, to develop useful diagnostic biomarkers enabling early diagnosis and, therefore, early effective treatment.

## 5. Conclusions

The role of EBV infection in the development of PCa has been a topic addressed by few researchers. However, it appears that EBV may be a contributing factor to the development and/or progression of PCa. Our results showed statistically significantly higher levels of antibodies, especially EBNA1, in more advanced clinical stages of PCa. We also observed a high viral load in patients with a high Gleason score. The results seem to be encouraging for further in-depth research in this area.

## Figures and Tables

**Table 1:** EUA risk groups for PCa [27,29].

Low Risk	Intermediate Risk	High Risk
PSA < 10 ng/mL	PSA 10–20 ng/mL	PSA > 20 ng/mL
GS < 7 (ISUP grade 1)	GS 7 (ISUP grade 2/3)	GS > 7 (ISUP grade 4/5)
cT1-2a	cT2b	cT2c

PSA: prostate-specific antigen and GS: Gleason score.

**Table 2:** Epidemiological and clinical characteristics of PCa patients.

		PCa Patients			
		EBV-Positive		EBV-Negative	
		*n*	%	*n*	%
Total		57	49.57	58	50.43
Age	54–59	7	12.28	11	18.97
	60–82	50	87.72	47	81.03
*p*		0.3239			
Place of residence	Urban	37	64.91	31	53.45
	Rural	20	35.09	27	46.55
*p*		0.2112			
Smoking	Never	12	21.05	10	17.24
	Ever	45	78.95	48	82.76
*p*		0.6034			
Alcohol abuse	Never	19	33.33	20	34.48
	≤drink per week	33	57.90	36	62.07
	>drink per week	5	8.77	2	3.45
*p*		0.4884			
Risk	Low	20	35.09	34	58.62
	Intermediate	13	22.81	16	27.59
	High	24	42.10	8	13.79
*p*		0.0026 *			
Gleason score	6	20	35.08	34	58.62
	7	13	22.81	16	27.59
	8	11	19.30	2	3.45
	9	13	22.81	6	10.34
*p*		0.0052 *			
T	T1	21	36.84	33	56.90
	T2	36	63.16	25	43.10
	T3	0	0.0	0	0.0
	T4	0	0.0	0	0.0
*p*		0.0312 *			
N	N0	57	100.0	58	100.0
M	M0	57	100.0	58	100.0

* Statistically significant; Pearson’s chi-squared test.

**Table 3:** Prevalence of EBVCA and EBNA1 antibodies in PCa patients according to the risk group.

		EBV-Positive				EBV-Negative		
	Low Risk *n* (%)	Intermediate Risk *n* (%)	High Risk *n* (%)	*p*	Low Risk *n* (%)	Intermediate Risk *n* (%)	High Risk *n* (%)	*p*
	*n* = 20	*n* = 13	*n* = 24		*n* = 34	*n* = 16	*n* = 8	
EBVCA IgA	12 (60.0)	10 (76.9)	22 (91.7)	0.0447 *	15 (38.2)	8 (50.0)	3 (37.5)	0.8379
EBVCA IgG	14 (70.0)	10 (76.9)	21 (87.5)	0.3585	14 (41.2)	10 (62.5)	4 (50.0)	0.8416
EBNA1 IgA	8 (40.0)	9 (69.2)	19 (79.2)	0.0240 *	13 (38.2)	7 (43.8)	4 (50.0)	0.8103
EBNA1 IgG	9 (45.0)	9 (69.2)	20 (83.3)	0.0265 *	13 (38.2)	7 (43.8)	3 (37.5)	0.9248

* Statistically significant; Pearson’s chi-squared test.

**Table 4:** Prevalence of EBVCA and EBNA1 antibodies in PCa patients according to GS.

	EBV-Positive					EBV-Negative				
	Gleason 6 *n* (%)	Gleason 7 *n* (%)	Gleason 8 *n* (%)	Gleason 9 *n* (%)	*p*	Gleason 6 *n* (%)	Gleason 7 *n* (%)	Gleason 8 *n* (%)	Gleason 9 *n* (%)	*p*
	*n* = 20	*n* = 13	*n* = 11	*n* = 13		*n* = 34	*n* = 16	*n* = 2	*n* = 6	
EBVCA IgA	12 (60.0)	10 (76.9)	10 (90.9)	12 (92.3)	0.1013	15 (44.1)	8 (50.0)	1 (50.0)	2 (33.3)	0.9140
EBVCA IgG	14 (70.0)	10 (76.9)	10 (90.9)	11 (84.6)	0.5332	20 (58.8)	10 (62.5)	0 (0.0)	4 (66.7)	0.3775
EBNA1 IgA	8 (40.0)	9 (69.2)	8 (72.7)	11 (84.6)	0.0499 *	13 (38.2)	7 (43.8)	0 (0.0)	4 (66.7)	0.3663
EBNA1 IgG	9 (45.0)	9 (69.2)	10 (90.9)	10 (76.9)	0.0506	13 (38.2)	7 (43.8)	1 (50.0)	2 (33.3)	0.9542

* Statistically significant; Pearson’s chi-squared test.

**Table 5:** Prevalence of EBVCA and EBNA1 antibodies in patients with PCa according to TNM classification (T feature).

	EBV-Positive			EBV-Negative		
	T1 *n* (%)	T2 *n* (%)	*p*	T1 *n* (%)	T2 *n* (%)	*p*
	*n* = 21	*n* = 36		*n* = 33	*n* = 25	
EBVCA IgA	13 (61.9)	31 (86.1)	0.0356 *	14 (42.4)	12 (48.0)	0.6724
EBVCA IgG	14 (66.7)	31 (86.1)	0.0824	20 (60.6)	14 (56.0)	0.7243
EBNA1 IgA	9 (42.9)	27 (75.0)	0.0152 *	12 (36.4)	12 (48.0)	0.3729
EBNA1 IgG	10 (47.6)	28 (77.8)	0.0198 *	13 (39.4)	10 (40.0)	0.9627

* Statistically significant; Pearson’s chi-squared test.

**Table 6:** EBV viral load in relation to GS in tumor tissue of patients with EBV-positive PCa.

Viral Load	Gleason Score		*p*
	Gleason 6–7 *n* (%)	Gleason 8–9 *n* (%)	
	*n* = 33	*n* = 24	
low	23 (69.7%)	8 (33.3%)	0.0083 *
high	10 (33.3%)	16 (66.7%)	

* Statistically significant; Pearson’s chi-squared test.

## Data Availability

Data are contained within the article.

## References

[1] World Health Organization Cancer Tomorrow. https://gco.iarc.fr/tomorrow/en/dataviz/isotype.

[2] Prostate Cancer: Statistics (2023). Approved by the Cancer.Net Editorial Board. https://www.cancer.net/cancer-types/prostate-cancer/statistics.

[3] World Health Organization (2020). The Global Cancer Observatory—All Rights Reserved. https://gco.iarc.fr/today/data/factsheets/cancers/27-Prostate-fact-sheet.pdf.

[4] Rawla P. (2019). Epidemiology of Prostate Cancer. World J. Oncol..

[5] Prostate Cancer—Is It Worth Getting Tested?. https://www.gov.pl.

[6] National Cancer Registry: Reports. https://onkologia.org.pl/pl/raporty.

[7] Pereira N.M., Martins E.A.C., Quintela M.G., Cunha A.A.D., Santos Netto M.M.D., Waisberg J. (2023). Presence of HPV in prostate tissue from patients submitted to prostate biopsy. Acta Cir. Bras..

[8] Leitzmann M.F., Rohrmann S. (2012). Risk factors for the onset of prostatic cancer: Age, location, and behavioral correlates. Clin. Epidemiol..

[9] Abidi S.H., Bilwani F., Ghias K., Abbas F. (2018). Viral etiology of prostate cancer: Genetic alterations and immune response. A literature review. Int. J. Surg..

[10] Gorish B.M.T., Ournasseir M.E.H., Shammat I.M. (2019). A correlation study of BK Polyoma Virus infection and prostate Cancer among Sudanese patients—Immunofluorescence and molecular based case-control study. Infect. Agents Cancer.

[11] Ge X., Wang X., Shen P. (2013). Herpes simplex virus type 2 or human herpesvirus 8 infection and prostate cancer risk: A meta-analysis. Biomed. Rep..

[12] Akram N., Imran M., Noreen M., Ahmed F., Atif M., Fatima Z., Bilal Waqar A. (2017). Oncogenic role of tumor viruses in humans. Viral Immunol..

[13] Moore P.S., Chang Y. (2010). Why do viruses cause cancer? Highlights of the first century of human tumour virology. Nat. Rev. Cancer.

[14] Kitsou K., Iliopoulou M., Spoulou V., Lagiou P., Magiorkinis G. (2021). Viral causality of human cancer and potential roles of human endogenous retroviruses in the multi-omics era: An evolutionary epidemiology review. Front. Oncol..

[15] Chakravorty S., Afzali B., Kazemian M. (2022). EBV-associated diseases: Current therapeutics and emerging technologies. Front. Immunol..

[16] IARC (1997). Proceedings of the IARC Working Group on the Evaluation of Carcinogenic Risks to Humans. Epstein-Barr Virus and Kaposi’s Sarcoma Herpesvirus/Human Herpesvirus 8. IARC Monographs on the Evaluation of Carcinogenic Risks to Humans.

[17] Akhtar S., Vranic S., Cyprian F.S., Al Moustafa A.E. (2018). Epstein-Barr Virus in Gliomas: Cause, Association, or Artifact?. Front. Oncol..

[18] Carneiro V.C.S., Pereira J.G., de Paula V.S. (2022). Family Herpesviridae and neuroinfections: Current status and research in progress. Mem. Inst. Oswaldo Cruz.

[19] Fugl A., Andersen C.L. (2019). Epstein-Barr virus and its association with disease—A review of relevance to general practice. BMC Fam. Pract..

[20] Sausen D.G., Bhutta M.S., Gallo E.S., Dahari H., Borenstein R. (2021). Stress-Induced Epstein-Barr Virus Reactivation. Biomolecules.

[21] Liu S., Zhao Z., Han L., Liu S., Luo B. (2016). Epstein-Barr Virus Infection in Gastric Remnant Carcinoma and Recurrent Gastric Carcinoma in Qingdao of Northern China. PLoS ONE.

[22] Chen X.Z., Che H., Castro F.H., Hu J., Brenner H. (2015). Epstein-Barr virus infection and gastric cancer: A systematic review. Medicine.

[23] Chen C.J., Hsu W.L., Yang H.I., Lee M.H., Chen H.C., Chien Y.C., You S.L. (2014). Epidemiology of virus infection and human cancer. Recent Results Cancer Res..

[24] Young L.S., Dawson C.W. (2014). Epstein-Barr virus and nasopharyngeal carcinoma. Chin. J. Cancer.

[25] De Lima M.A.P., Teodoro I.P.P., Galiza L.E., Filho P.H.B.M., Marques F.M., Pinheiro Junior R.F.F., Macedo G.E.C., Facundo H.T., da Silva C.G.L., Lima M.V.A. (2019). Association between Epstein-Barr Virus and Oral Carcinoma: A Systematic Review with Meta-Analysis. Crit. Rev. Oncog..

[26] Ahmed K., Sheikh A., Fatima S., Haider G., Ghias K., Abbas F., Mughal N., Abidi S.H. (2022). Detection and characterization of latency stage of EBV and histopathological analysis of prostatic adenocarcinoma tissues. Sci. Rep..

[27] Epstein J.I., Egevad L., Amin M.B., Delahunt B., Srigley J.R., Humphrey P.A., Grading Committee (2016). The 2014 International Society of Urological Pathology (ISUP) Consensus Conference on Gleason Grading of Prostatic Carcinoma: Definition of Grading Patterns and Proposal for a New Grading System. Am. J. Surg. Pathol..

[28] Epstein J.I., Zelefsky M.J., Sjoberg D.D., Nelson J.B., Egevad L., Magi-Galluzzi C., Vickers A.J., Parwani A.V., Reuter V.E., Fine S.W. (2016). A Contemporary Prostate Cancer Grading System: A Validated Alternative to the Gleason Score. Eur. Urol..

[29] EAU Guidelines for Prostate Cancer. https://uroweb.org/guidelines/prostate-cancer/chapter/classification-and-staging-systems.

[30] Bertero L., Massa F., Metovic J., Zanetti R., Castellano I., Ricardi U., Papotti M., Cassoni P. (2018). Eighth Edition of the UICC Classification of Malignant Tumours: An overview of the changes in the pathological TNM classification criteria-What has changed and why?. Virchows Arch..

[31] De Bono J.S., Guo C., Gurel B., De Marzo A.M., Sfanos K.S., Mani R.S., Gil J., Drake C.G., Alimonti A. (2020). Prostate carcinogenesis: Inflammatory storms. Nat. Rev. Cancer.

[32] Göbel A., Dell’Endice S., Jaschke N., Pählig S., Shahid A., Hofbauer L.C., Rachner T.D. (2021). The role of inflammation in breast and prostate cancer metastasis to bone. Int. J. Mol. Sci..

[33] Lawson J.S., Glenn W.K. (2022). Multiple pathogens and prostate cancer. Infect. Agents Cancer.

[34] Sfanos K.S., Sauvageot J., Fedor H.L., Dick J.D., De Marzo A.M., Isaacs W.B. (2008). A molecular analysis of prokaryotic and viral DNA sequences in prostate tissue from patients with prostate cancer indicates the presence of multiple and diverse microorganisms. Prostate.

[35] Nahand J.S., Khanaliha K., Mirzaei H., Moghoofei M., Baghi H.B., Esghaei M., Khatami A.R., Fatemipour M., Bokharaei-Salim F. (2021). Possible role of HPV/EBV coinfection in anoikis resistance and development in prostate cancer. BMC Cancer.

[36] Whitaker N.J., Glenn W.K., Sahrudin A., Orde M.M., Delprado W., Lawson J.S. (2013). Human papillomavirus and Epstein Barr virus in prostate cancer: Koilocytes indicate potential oncogenic influences of human papillomavirus in prostate cancer. Prostate.

[37] Grinstein S., Preciado M.V., Gattuso P., Chabay P.A., Warren W.H., De Matteo E., Gould V.E. (2002). Demonstration of Epstein-Barr virus in carcinomas of various sites. Cancer Res..

[38] Bergh J., Marklund I., Gustavsson C., Wiklund F., Grönberg H., Allard A., Alexeyev O., Elgh F. (2007). No link between viral findings in the prostate and subsequent cancer development. Br. J. Cancer..

[39] Szkaradkiewicz A., Wal M., Kuch A., Pieta P. (2004). Human papillomavirus (HPV) and Epstein-Barr virus (EBV) cervical infections in women with normal and abnormal cytology. Pol. J. Microbiol..

[40] Huang W.Y., Hayes R., Pfeiffer R., Viscidi R.P., Lee F.K., Wang Y.F., Reding D., Whitby D., Papp J.R., Rabkin C.S. (2008). Sexually transmissible infections and prostate cancer risk. Cancer Epidemiol. Prevent. Biomark..

[41] Tsai M.H., Raykova A., Klinke O., Bernhardt K., Gärtner K., Leung C.S., Geletneky K., Sertel S., Münz C., Delecluse H.J. (2013). Spontaneous lytic replication and epitheliotropism define an Epstein-Barr virus strain found in carcinomas. Cell Rep..

[42] Young L.S., Yap L.F., Murray P.G. (2016). Epstein-Barr virus: More than 50 years old and still providing surprises. Nat. Rev. Cancer.

[43] Cohen J.I. (2000). Epstein-Barr virus infection. N. Engl. J. Med..

[44] Kikuchi K., Noguchi Y., de Rivera M.W., Hoshino M., Sakashita H., Yamada T., Inoue H., Miyazaki Y., Nozaki T., González-López B.S. (2016). Detection of Epstein-Barr virus genome and latent infection gene expression in normal epithelia, epithelial dysplasia, and squamous cell carcinoma of the oral cavity. Tumour Biol..

[45] Kang M.S., Kieff E. (2015). Epstein-Barr virus latent genes. Exp. Mol. Med..

[46] De Paschale M., Clerici P. (2012). Serological diagnosis of Epstein-Barr virus infection: Problems and solutions. World. J. Virol..

[47] Sinha S., Dickey B.L., Coghill A.E. (2022). Utility of Epstein-Barr virus (EBV) antibodies as screening markers for nasopharyngeal carcinoma: A narrative review. Ann. Nasopharynx Cancer.

[48] Chien Y.C., Chen J.Y., Liu M.Y., Yang H.I., Hsu M.M., Chen C.J., Yang C.S. (2001). Serologic markers of Epstein-Barr virus infection and nasopharyngeal carcinoma in Taiwanese men. N. Engl. J. Med..

[49] Liu W., Chen G., Gong X., Wang Y., Zheng Y., Liao X., Liao W., Song L., Xu J., Zhang X. (2021). The diagnostic value of EBV-DNA and EBV-related antibodies detection for nasopharyngeal carcinoma: A meta-analysis. Cancer Cell Int..

[50] Sun K., Jia K., Lv H., Wang S.Q., Wu Y., Lei H., Chen X. (2020). EBV-Positive Gastric Cancer: Current Knowledge and Future Perspectives. Front. Oncol..

[51] Sivachandran N., Wang X., Frappier L. (2012). Functions of the Epstein-Barr virus EBNA1 protein in viral reactivation and lytic infection. J. Virol..

[52] Li H., Liu S., Hu J., Luo X., Li N., Bode A.M., Cao Y. (2016). Epstein-Barr virus lytic reactivation regulation and its pathogenic role in carcinogenesis. Int. J. Biol. Sci..

[53] Lo A.K., Dawson C.W., Lung H.L., Wong K.L., Young L.S. (2020). The Therapeutic Potential of Targeting BARF1 in EBV-Associated Malignancies. Cancers.

[54] Hoebe E.K., Le Large T.Y., Greijer A.E., Middeldorp J.M. (2013). BamHI-A rightward frame 1, an Epstein-Barr virus-encoded oncogene and immune modulator. Rev. Med. Virol..

[55] Zur Hausen A., Brink A.A., Craanen M.E., Middeldorp J.M., Meijer C.J., van den Brule A.J. (2000). Unique transcription pattern of Epstein–Barr virus (EBV) in EBV-carrying gastric adenocarcinomas: Expression of the transforming BARF1 gene. Cancer Res..

[56] Zheng H., Li L.L., Hu D.S., Deng X.Y., Cao Y. (2007). Role of Epstein–Barr virus encoded latent membrane protein 1 in the carcinogenesis of nasopharyngeal carcinoma. Cell. Mol. Immunol..

